# Diagnostic performance of GAAD, PIVKA-II and AFP in hepatocellular carcinoma, chronic liver disease and healthy population: evidence from a Malaysian cohort

**DOI:** 10.1186/s12876-026-05140-6

**Published:** 2026-07-25

**Authors:** Rabiatul Adawiyyah Mohamad Noor, Nur Karyatee Kassim, Aniza Mohammed Jelani, Lee Yeong Yeh, Mohd Hafizuddin Husin, Wan Muhamad Amir W Ahmad, Nurhanis Syazni Roslan

**Affiliations:** 1 Department of Chemical Pathology, School of Medical Sciences, Health Campus, Universiti Sains Malaysia, Kota Bharu, Kelantan Malaysia; 2 School of Dental Sciences, Health Campus, Universiti Sains Malaysia, Kota Bharu, Kelantan Malaysia; 3 Department of Internal Medicine, School of Medical Sciences, Health Campus, Universiti Sains Malaysia, Kota Bharu, Kelantan Malaysia; 4 Department of Radiology, School of Medical Sciences, Health Campus, Universiti Sains Malaysia, Kota Bharu, Kelantan Malaysia; 5 Department of Medical Education, School of Medical Sciences, Health Campus, Universiti Sains Malaysia, Kota Bharu, Kelantan Malaysia; 6 Hospital Pakar Universiti Sains Malaysia, Health Campus, Universiti Sains Malaysia, Kota Bharu, Kelantan Malaysia; 7 Department of Pathology, Hospital Sultanah Nur Zahirah, Terengganu, Malaysia

**Keywords:** AFP, CLD, HCC, PIVKA-II, GAAD

## Abstract

**Background:**

The aim of the current study was to evaluate the diagnostic performances of GAAD score, PIVKA-II and alpha-fetoprotein (AFP) for hepatocellular carcinoma (HCC) surveillance in HCC, chronic liver disease (CLD) and healthy controls (HC) subjects from the northeastern Peninsular Malaysia.

**Methods:**

This was a single-center cross-sectional study. The GAAD value was calculated using the Elecsys® GAAD calculator. Serum PIVKA-II and AFP levels were measured using the Elecsys® electrochemiluminescence immunoassay method. Diagnostic performance of these tests were evaluated based on area under the curve (AUC), optimal cutoff levels, sensitivity (Se), specificity (Sp), positive predictive value (PPV) and negative predictive value (NPV).

**Results:**

Of the 128 enrolled subjects,99 were included (33 HCC, 33 CLD, and 33 HC). GAAD score, PIVKA-II, and AFP levels were significantly higher in patients with HCC than in those with CLD and HC (all *p* < 0.05). For differentiating HCC from CLD, GAAD at a cutoff of 2.57 demonstrated the highest Se (81.8%) and Sp (72.7%), with an AUC of 0.873 (95% CI 0.791–0.955). PIVKA-II at a cut-off of 28.4 ng/mL showed Se and Sp of 75.8% and 69.7%, respectively, with an AUC of 0.842 (95% CI 0.748–0.936). AFP at a cutoff of 20 ng/mL demonstrated lower Se (51.5%) but higher Sp (90.9%), with an AUC of 0.834 (95% CI 0.730–0.938). For differentiating HCC from HC, GAAD and PIVKA-II achieved 100% Sp and PPV, whereas AFP maintained 100% Sp but lower Se (51.52%). Overall, GAAD exhibited the best balance of sensitivity and specificity for HCC detection, while PIVKA-II provided complementary diagnostic value due to its high specificity.

**Conclusion:**

The GAAD score demonstrated superior diagnostic performance compared with both PIVKA-II and AFP in differentiating HCC from CLD and HC subjects, supporting its potential utility in HCC surveillance. In addition, PIVKA-II showed better diagnostic performance than AFP.

## Introduction

The global incidence of hepatocellular carcinoma (HCC) is increasing, presenting a significant concern for global health [1, 2]. The World Health Organization estimates that HCC is the third leading cause of cancer-related mortality and the sixth-most common malignancy worldwide [3, 4]. According to Singal et al. [5], Asia accounts for 72% HCC cases with more than half occurring in China, followed by Europe (10%) and Africa (7.8%) [5]. In Malaysia, the incidence rose to 6.1% in 2013 [6]. Hepatitis B virus (HBV) infection is the leading cause of HCC in Malaysia, followed by cryptogenic, metabolic dysfunction-associated fatty liver disease (MAFLD), and metabolic dysfunction-associated steatotic liver disease (MASLD). Despite advances in imaging and therapeutic approaches, early diagnosis remains the most important determinant of curative treatment and long-term survival [2].

Currently, imaging-based surveillance, particularly abdominal ultrasonography, remains the cornerstone of HCC surveillance among individuals at high risk because of its non-invasive nature, wide availability, and ability to detect focal liver lesions requiring further evaluation. Nevertheless, the sensitivity of ultrasonography may be influenced by tumour size, liver cirrhosis, operator expertise, and patient-related factors, highlighting the need for complementary serum biomarkers to improve early HCC detection [7]. The Asian Pacific Association for the Study of the Liver (APASL), the European Association for the Study of the Liver (EASL), and the American Association for the Study of Liver Diseases (AASLD) recommend monitoring for HCC in patients with cirrhosis and those at high-risk due to hepatitis B [8]. Hepatic ultrasonography (US) and serum AFP are the most commonly ordered surveillance tests. The AASLD recommends six monthly surveillances with or without AFP [9]. While the US screening method is the most cost-effective, it has limitation in sensitivity (range 30–70%), and it is operator-dependent, meaning a negative result does not definitely exclude the presence of HCC [10, 11]. Combining AFP with ultrasound at 6-month intervals is an alternative screening method to ultrasound alone. This combination has a sensitivity of 63% for detecting early-stage HCC, 97% for diagnosing HCC at any stage and a specificity of 84% [12, 13].

Traditionally, serum AFP has been widely used as a biomarker for benign and malignant liver diseases. Elevated AFP levels have been observed in various benign liver disorders, including 15- 46% of HCV infection cases and 11–47% of liver cirrhosis [14]. AFP levels are also elevated in 60–80% of cases using a diagnosis cutoff level of 20 ng/mL [15]. However, approximately 30% of HCC cases are AFP-negative, leading to a delayed diagnosis [8, 16, 17]. Due to its low sensitivity, there is an urgent need for an alternative biomarker to bridge this diagnostic gap.

Prothrombin induced by vitamin K absence or antagonist-II (PIVKA-II), also known as des-carboxy-prothrombin (DCP), is an emerging biomarker for HCC diagnosis currently being studied. Liebman et al. (1984) discovered that PIVKA-II is significantly elevated in the serum of HCC patients, suggesting its potential as a serum biomarker for the disease [18]. PIVKA-II is a prothrombin molecule that is abnormally synthesized as the result of an acquired flaw in the prothrombin precursor's posttranslational carboxylation produced by malignant hepatocytes (Liebman et al. 1984). Studies indicate that PIVKA II may outperformed AFP in diagnostic accuracy, and combining both markers enhances diagnostic performance in HCC surveillance [18–21].

The gender, age, AFP, des-gamma carboxy-prothrombin (DCP) algorithm has recently emerged as a valuable tool for HCC surveillance in high-risk patients. Developed by Roche Diagnostics International Ltd in Rotkreuz, Switzerland, the GAAD score, is an in vitro diagnostic multivariate index assay [22]. This algorithm integrates several criteria including gender, age, AFP, and PIVKA-II, to generate a semi-quantitative result, on a scale of 1 to 10.

To date, only a few studies have investigated the clinical utility of GAAD score in assessing patients with HCC and CLD. In Taiwan, a study concluded that PIVKA-II and GAAD score are more sensitive than AFP in detecting HCC among CLD patients and GAAD score might outperform PIVKA-II, particularly in detecting early HCC cases [23]. The Japan Society of Hepatology currently endorses PIVKA-II as a tumor marker for surveillance of hepatocellular carcinoma [24]. Moreover, the Asia–Pacific expert consensus recognized that PIVKA-II is a valuable biomarker for the detection of HCC in AFP-negative patients, as well as for treatment monitoring and the prediction of microvascular invasion in transplant candidates [25].

Currently, the utilization of AFP for HCC surveillance remains suboptimal, and the diagnostic performance of the emerging biomarker PIVKA-II continues to be evaluated given the influences of ethnic and geographic factors on biomarker expression. As a results, the diagnostic findings observed in Western or East Asian populations may not fully reflect those within the Malaysia population. To address this gap, the present study aims to assess the diagnostic performance of GAAD score, PIVKA-II and AFP in Malaysian clinical setting, where evidence remains limited. Furthermore, GAAD score has shown promise as a sensitive tool for the early detection of HCC. Through this investigation, we aim to provide population-specific insights that may enhance decision making and improve the effectiveness of HCC surveillance strategies in Malaysia.

## Material & methods

This was a cross-sectional study involving subjects with HCC and healthy volunteers recruited from Hospital Pakar Universiti Sains Malaysia, a tertiary referral centre located in the northeast region of Peninsular Malaysia between December 2023 and December 2024. The minimum sample size was calculated using G*Power software, with an effect size of 0.4, 80% statistical power, and 95% confidence interval (CI) yielding a total of 99 participants after allowing for a 10% adjustment [26]. To ensure balanced comparison among the three study groups, eligible participants were consecutively recruited until each group comprised 33 individuals: HCC, chronic liver disease and healthy controls. All 99 recruited participants fulfilled the eligibility criteria and had complete clinical and laboratory data available for final analysis. Therefore, no participants were excluded from the final dataset. Participants were recruited consecutively according to the predefined inclusion and exclusion criteria, thereby minimising selection bias.

The study protocol was reviewed and approved by the Human Research Ethics Committee of University Sains Malaysia (USM/JEPeM/18010058) and in line with the Helsinki Declaration. All subjects signed the informed consent form for the study.

### HCC subjects

The inclusion criteria for HCC subjects included those aged 18 years old and above, with a confirmed diagnosis of HCC clinically and radiologically as recommended by previous studies [10]. The computed tomography (CT) scan finding should include evidence of early washout in the portal phase and enhancement in the arterial phase. Additional clinicopathological characteristics were recorded including the number of tumours, their diameter at the longest axis, the involvement of portal vein thrombosis, and the presence of metastases. The largest tumour size was measured if several tumours were detected. Demographic data and risk factors associated with HCC were also gathered.

### CLD subjects

Eligible CLD subjects were those aged at least 18 years old and diagnosed with CLD for six months or longer and was confirmed by ultrasound findings such as nodular surface, coarse and heterogeneous echo texture, segmental hypertrophy/atrophy, signs of portal hypertension and the presence of splenomegaly/ascites/fatty changes. The etiologies of CLD were recorded, such as cirrhosis, HBV & HCV infection, MAFLD, and others.

### Healthy subjects

Eligible healthy subjects were 18 years old and above, with no prior history of chronic medical illnesses, no history of liver disease, no risk factors for viral hepatitis, and normal liver biochemistry. The subjects were volunteers recruited within the hospital compound including staffs and family members through advertisement.

Exclusion criteria were those diagnosed with liver metastases and other hepatic tumours besides HCC, gallstone, cholangiocarcinoma, cancer of the head of pancreas, obstructive jaundice, as well as being on warfarin or vitamin K six months before testing.

### Specimen collection and analysis

#### Determination of GAAD score

Elecsys® GAAD score was calculated using Roche diagnostic proprietary formulas, which included inserting Elecsys® PIVKA, Elecsys® AFP, age and gender at birth as input parameters. The manufacturer’s reference cutoff for the GAAD score (Z) is 2.57; a test score ≥ 2.57 is indicative of a positive result and denotes a high risk for HCC, thereby necessitating further diagnostic evaluation [22].

#### Measurement of PIVKA-II and AFP level

Peripheral blood samples were collected before the commencement of treatment of HCC. The samples were centrifuged and stored at −80 °C until analysis. Serum levels of PIVKA-II and AFP were measured using the Roche Cobas e601 System (Elecsys®, ROCHE Diagnostics) according to the manufacturer’s instructions. Both assays were performed based on a two-step immunoassay technique. The quantitative measurement of serum PIVKA-II was based on the electro-chemiluminescence immunoassay (ECLIA) technology using the sandwich principle. The established cutoff value for PIVKA-II in HCC diagnosis was 28.4 ng/mL, whereas AFP cutoff value for the early detection of HCC was set at 20 ng/mL [23].

### Data and statistical analysis

Data was entered and analyzed using IBM SPSS software Version 27.0 (SPSS Inc., Chicago, IL, USA). Numerical data were expressed as mean and standard deviation (SD) unless otherwise mentioned. Categorical data were presented as frequency and percentages. The Kruskal Wallis test was employed to compare non-parametric continuous variables followed by the Mann–Whitney U test for post hoc tests. The receiver operating characteristic (ROC) curve analysis was conducted to evaluate the diagnostic performance of the GAAD score, PIVKA-II, and AFP. The area under the ROC curve (AUC) with corresponding 95% confidence interval (CI) was calculated, using all possible threshold values. Sensitivity, specificity, positive predictive value (PPV), and negative predictive value (NPV) were subsequently determined at predefined cutoff values based on established clinical thresholds and previous literature. A *p*-value of < 0.05 was considered statistically significant.

## Results

### Baseline characteristics of study subjects

Of 128 subjects screened, 33 HCC, 33 CLD, and 33 HC subjects were enrolled in the study. Twenty-nine screened subjects (16 HCC, 4 CLD, and 9 HC subjects) were excluded due to incomplete sample processing or based on investigator’s clinical decision.

The baseline demographic characteristics and biochemical laboratory profiles of the study populations were shown in Tables 1 and 2. The mean (SD) age of HCC participants was 66.4 (8.8) years, with a male predominance (n = 33,85%). In comparison, the CLD and HC subjects had mean ages of 59.9 (12.8) years and 46.7(9.8) years, respectively, both with equal gender distribution. HBV infection was the leading cause of HCC (n = 18, 55%); followed by HCV infection (n = 5, 15%) and MAFLD (n = 4,12%). A similar pattern was observed among the CLD subjects, where HBV infection was the predominant underlying cause (n = 18,55%), followed by MAFLD (n = 6, 18%) and HCV infection (n = 4,12%).

**Table 1:** Demographic profile of HCC, CLD, and HC subjects

	**HCC,n = 33** **n (%)**	**CLD,n = 33** **n (%)**	**HC,n = 33** **n (%)**
**Age, year**			
* Mean (SD****)***	66.4(8.8)	59.9(12.8)	46.7(9.8)
**Gender**			
*Male*	28(85)	17(52)	16(48)
*Female*	5(15)	16(48)	17(52)
**Race**			
*Malay*	28(85)	31(94)	31(94)
*Non-Malay*	5(15)	2(6)	2(6)
**Etiology**			
*HBV infection*	18(55)	18(55)	-
*HCV infection*	5(15)	4(12)	-
*MAFLD*	4(12)	6(18)	-
*AIH*	0(0)	2(6)	-
*Drugs*	1(3)	1(3)	-
*Others*	6(18)	2(6)	-
**Clinicopathological **			
**Features**			
*Tumour number(nodule)*-			
*Single*	13(39)		-
*Multiple*	20(60)	-	-
*Tumour size*			
< *3 cm*	10(30)		
*3–5 cm*	5(15)		
> *5 cm*	18(54)	-	-
*Portal vein thrombosis*		-	
*Present*	5(15)		-
*Absent*	28(85)	-	-
*Metastasis*			
*Yes*	1(3)		
*No*	32(97)	-	-

*HCC* Hepatocellular Carcinoma, *CLD* Chronic Liver Disease, *HC* Healthy Control, *HBV* Hepatitis B Virus, *HCV* Hepatitis C Virus, *MAFLD* Metabolic Associated Fatty Liver Disease, *AIH* Autoimmune Hepatitis

**Table 2:** Biochemical parameters among HCC, CLD, and HC subjects

PARAMETER Median (IQR)	HCC	CLD	HC
*Total bilirubin (umol/L)*	23(22)	18(20)	4(4)
*Albumin (g/L)*	33(9)	36(9)	46(5)
*AST (U/L)*	77(118)	40(24)	21(8)
*ALT (U/L)*	62(107)	28(20)	17(11)
*ALP (U/L)*	143(127)	135(108)	71(23)

*IQR* Interquartile Range, *HCC* Hepatocellular Carcinoma, *CLD* Chronic Liver Disease, *HC* Healthy Control, *AST* Aspartate Transaminase, *ALT* Alanine Transferase, *ALP* Alkaline Phosphatase

Based on clinicopathologic characteristics, 60% (n = 20) of HCC patients presented with multiple nodules, while 54% (n = 18) had tumours measuring more than 5 cm at baseline. Portal vein thrombosis was identified in 15% of cases (n = 5).

### Comparison of the level of GAAD score, PIVKA-II and AFP among HCC, CLD, and HC subjects

The GAAD score, PIVKA-II and AFP levels were significantly higher in HCC subjects compared to the CLD and HC subjects (*p* < 0.05) (Table 3). The median (IQR) of GAAD score for HCC, CLD, and HC subjects were 9.59 (5.37), 1.2 (2.74), and 0.21(0.39), respectively. Correspondingly, median (IQR) PIVKA-II levels demonstrated a similar pattern, with markedly elevated levels in the HCC subjects at 197.3 (2864.9) ng/ml, compared with 21.1(31.1) ng/ml in the CLD subjects, and 16.68 (3.59) ng/ml in the HC subjects. Similarly, the median (IQR) for AFP levels were 20.0 (767.5) ng/ml in the HCC group, 4.3(3.2) ng/ml in the CLD group, and 2.2 (1.4) ng/ml in HC subjects.

**Table 3:** Comparison of the level of PIVKA-II and AFP among HCC, CLD, and HC subjects

Biomarkers	Median (IQR)				
	HCC	CLD	HC	*x* ^2^ statistic(df)^a^	*p*-value
GAAD	9.59(5.37)	1.2(2.74)	0.21(0.39)	59.0(2)	< 0.05
*PIVKA-II (ng/ml)*	197.3(2864.9)	21.1(31.1)	16.68(3.59)	48.5(2)	< 0.05
*AFP* *(ng/ml)*	20.0(767.5)	4.2(3.2)	2.2(1.7)	44.7(2)	< 0.05

*p*-value with a significance level of < 0.05

PIVKA-II: Post hoc multiple comparison test HCC vs CLD, *p* =.000; HCC vs HC, *p* =.000; CLD vs HC, *p* =.017 (Bonferroni correction of *p*-value)

AFP: Post hoc multiple comparison test HCC vs CLD, *p* =.000; HCC vs HC, *p* =.000; CLD vs HC, *p* =.002 (Bonferroni correction of *p*-value)

*HCC* Hepatocellular Carcinoma, *CLD* Chronic Liver Disease, *HC* Healthy Control, *PIVKA-II* Protein Induced by Vitamin-K Absence-II, *AFP* Alpha-Fetoprotein, *IQR* Interquartile Range

a = Statistical test: Kruskal–Wallis test, applied for non-parametric and non-normal distributed data

### Diagnostic performance of GAAD score, PIVKA-II and AFP between HCC and CLD subjects

**Fig. 1:**
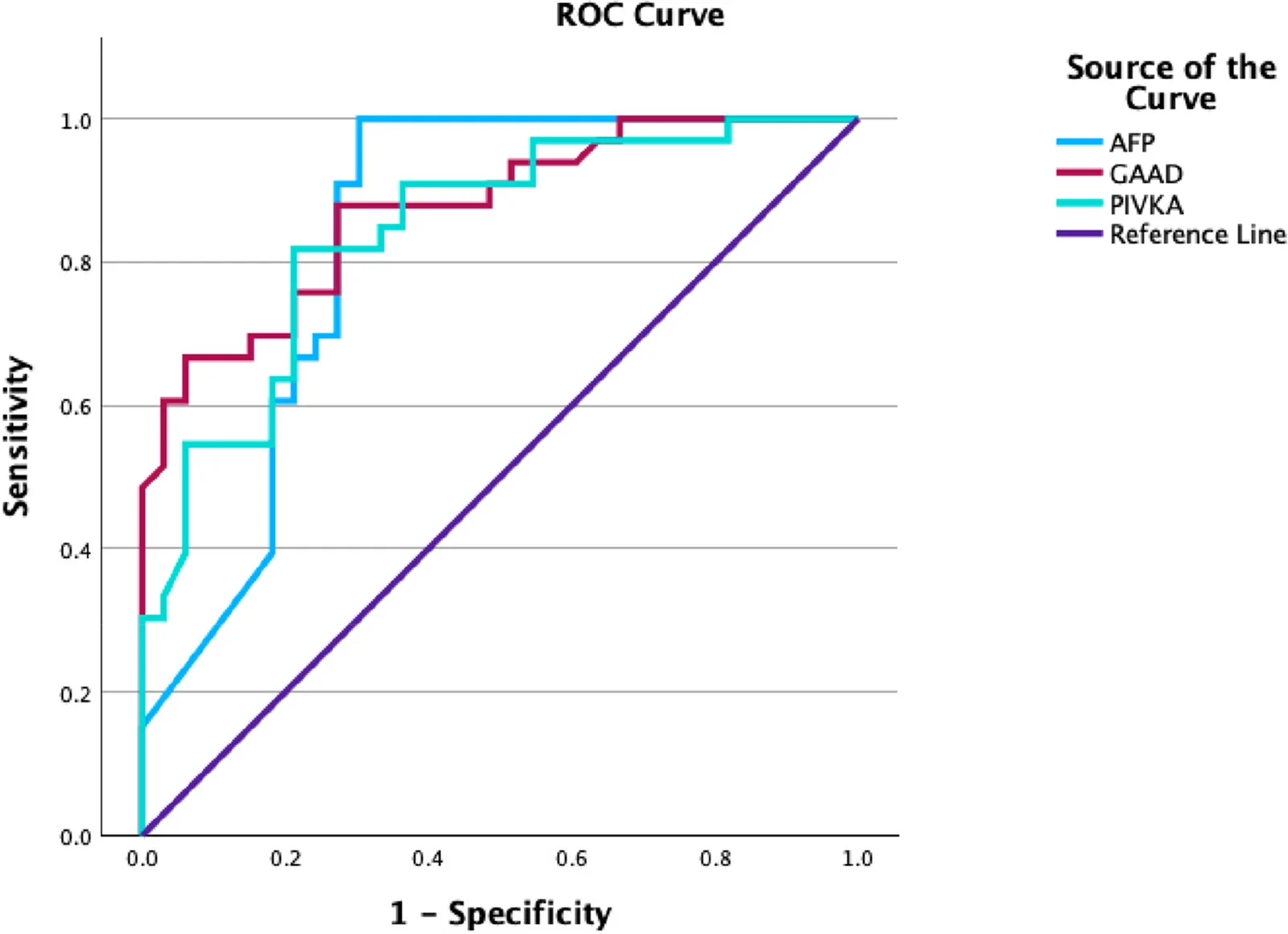
ROC of GAAD,PIVKA-II and AFP for diagnosis of HCC in HCC versus CLD subjects

As shown in Fig. 1 and Table 4, the GAAD score demonstrated the highest diagnostic accuracy in differentiating HCC from CLD subjects, with an AUC of 0.87 (95%CI 0.80–0.96), followed by PIVKA-II [AUC 0.84 (95% CI 0.75–0.94)] and AFP [0.83 (95%CI 0.73–0.94]. The optimal cutoff value for the GAAD score was determined using Youden index and was set at 2.57. At this cutoff, the GAAD score demonstrated a sensitivity of 81.8%, specificity of 72.7%, positive predictive value (PPV) of 75.0%, and negative predictive value (NPV) of 80.0%.

**Table 4:** Diagnostic performance of GAAD,PIVKA-II and AFP for diagnosis of HCC in HCC and HC subjects

Variables	AUC (95%CI)	Cutoff value	Sn(%) (95%CI)	Sp(%) (95%CI)	PPV(%) (95%CI)	NPV(%) (95%CI)
*GAAD*	0.873 (0.791–0.955)	*2.57*	81.82 (0.66–0.91)	72.73 (0.56–0.85)	75.00 (0.59–0.86))	80.0 (0.63–0.91)
*PIVKA-II*	0.842 (0.748–0.936)	28.4	75.76 (0.59–0.87)	69.70 (0.53–0.83)	71.43 (0.55–0.84)	74.19 (0.57–0.86)
*AFP*	0.834 (0.730–0.938)	20	51.52 (0.35–0.66)	90.90 (0.76–0.97)	85.00 (0.64–0.95)	65.22 (0.51–0.77)

*AUC* area under curve, *Sn* Sensitivity, *Sp* Specificity, *PPV* positive predictive value, *NPV* negative predictive value, *HCC* Hepatocellular carcinoma, *CLD* Chronic liver disease, *PIVKA-II* Protein Induced by Vitamin-K Absence-II, *AFP* Alpha-fetoprotein

Similarly, the optimal cutoff level for PIVKA-II, identified using the Youden index, was 28.4 ng/ml, yielding a sensitivity of 75.8%, specificity of 69.7%, PPV of 71.4%, and an NPV of 74.2%. For AFP cutoff level of 20 ng/ml resulted in a lower sensitivity of 51.52% but higher specificity at 90.9%, with a PPV of 85.0% and NPV of 65.2%.

### Diagnostic performance of GAAD score, PIVKA-II and AFP between HCC and HC subjects

**Fig. 2:**
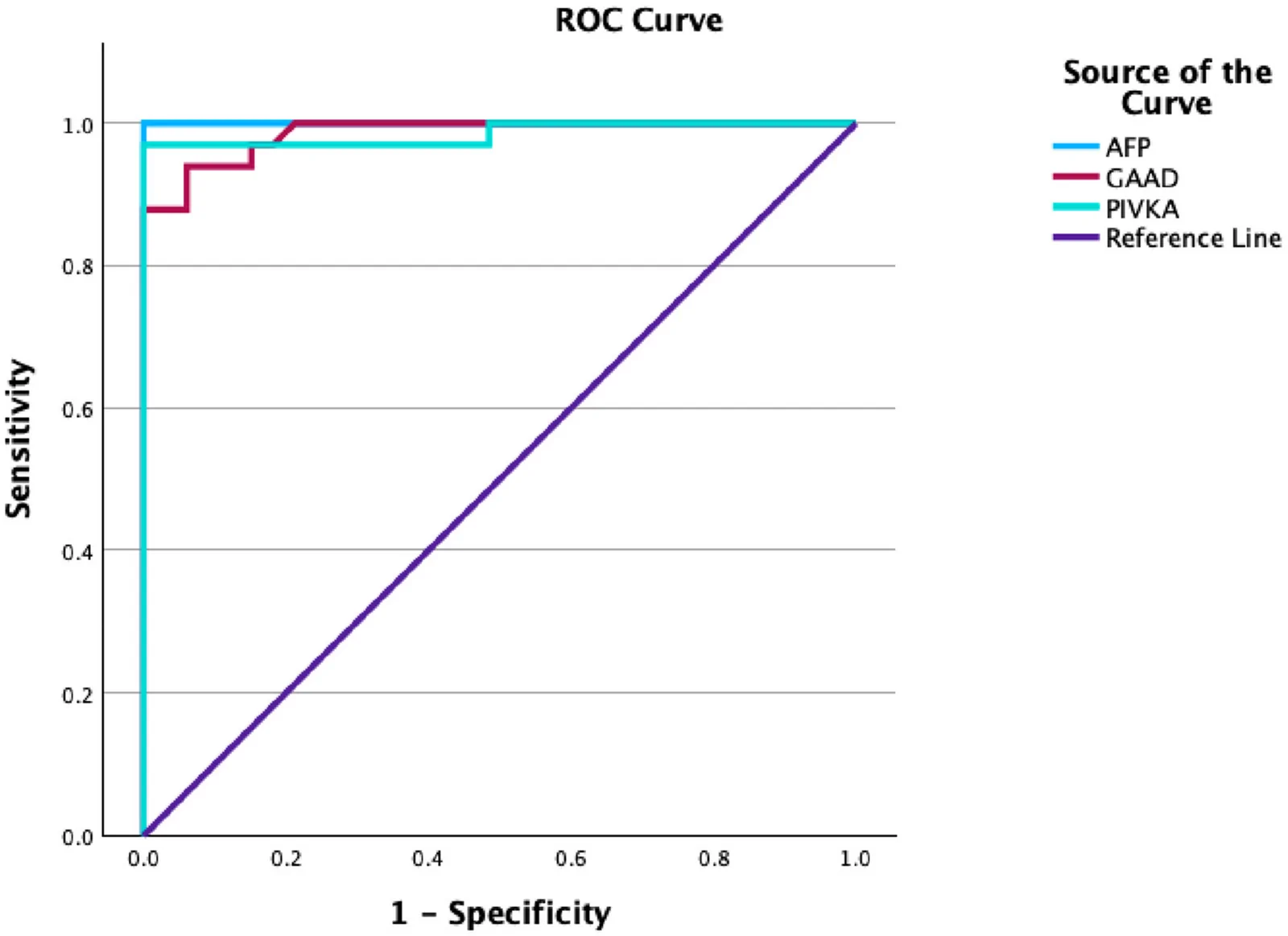
ROC of GAAD, PIVKA-II and AFP for diagnosis of HCC in HCC versus HC subjects

Figure 2 illustrates ROC curves for the GAAD score, PIVKA-II and AFP in distinguishing HCC from HC subjects. All three biomarkers demonstrated strong discriminative ability, with AUC values indicating excellent diagnostic performance. The GAAD score exhibited higher diagnostic accuracy than PIVKA-II with AUC values of 0.986 (95% CI 0.97–1.00) and 0.985 (95% CI 0.956–1.00)] respectively. AFP demonstrated the highest diagnostic performance with an AUC of 1.00 (95% CI 1.00–1.00).

Using the standard cutoff level of 2.57, the GAAD score differentiated HCC from HC subjects with a highest sensitivity of 81.8%, a specificity of 100%, PPV of 100%, and NPV of 84.6% (Table 5). At its optimal cutoff level of 28.4 ng/mL, PIVKA-II demonstrated a 75.8% sensitivity, a 100% specificity, a 100% PPV and an NPV of 80.5%. For AFP, the optimal cutoff level of 20 ng/ml showed a lower sensitivity at 51.5%, but retained a high specificity of 100%, with a PPV of 100% and NPV of 67.4%.

**Table 5:** Diagnostic performance of GAAD, PIVKA-II and AFP for diagnosis of HCC in HCC and HC subjects

Variables	AUC (95%CI)	Cutoff value	Sn(%) (95%CI)	Sp(%) (95%CI)	PPV(%) (95%CI)	NPV(%) (95%CI)
*GAAD*	0.986 (0.97–1.0)	*2.57*	81.82 (0.66–0.91)	100.0 (0.90–1.0)	100 (0.88–1.0)	84.62 (0.70–0.92)
*PIVKA-II*	0.985 (0.96–1.0)	28.4	75.76 (0.59–0.87)	100.0 (0.90–1.0)	100 (0.87–1.0)	80.48 (0.66–0.90)
*AFP*	1.00 (1.0–1.0)	20.0	51.52 (0.35–0.68)	100.0 (0.90–1.0)	100 (0.83–1.0)	67.35 (0.53–0.79)

*AUC* area under curve, *Sn* Sensitivity, *Sp* Specificity, *PPV* positive predictive value, *NPV* negative predictive value, *HCC* Hepatocellular carcinoma, *HC* Healthy control, *PIVKA-II* Protein Induced by Vitamin-K Absence-II, *AFP* Alpha-fetoprotein

## Discussion

We have evaluated the diagnostic performance of the GAAD score in comparison with PIVKA-II and AFP within a Malaysian cohort, providing population-specific evidence on the advantages of integrating multiple biomarkers with demographic risk factors.

The demographic characteristics reflect known epidemiological patterns of HCC in the Malaysian population. Subjects with HCC were older and mostly male compared with CLD and HC subjects, and hepatitis B infection was the dominant etiology, all consistent with other global and regional data [6, 27–30].

The median GAAD scores obtained in our cohort of 1.2 in CLD, and 0.21 in HC subjects were largely consistent with published values which generally ranged between 0.33–1.78. However, the GAAD scores (9.59) observed for HCC were higher than those reported in several other studies, where median scores ranged from 5.05 to 6.6 [23, 31]. It is unclear the reasons for this variation but could be multifactorial including population-specific factors, different liver disease profile or different tumour burden, and of course sample sizes. An important consideration when interpreting the present findings is the clinical stage of the included HCC patients. More than half of the patients presented with tumours larger than 5 cm, while approximately 60% had multifocal disease, reflecting the case mix commonly encountered in our tertiary referral centre. Because circulating concentrations of AFP and PIVKA-II generally correlate with tumour burden, the predominance of advanced-stage HCC may have contributed to the excellent diagnostic performance observed for both individual biomarkers and the composite GAAD score. Consequently, the diagnostic sensitivity reported in the present study may represent an optimistic estimate compared with that expected in surveillance populations, where the principal objective is the detection of small, early-stage HCC.

Similarly, the medians of PIVKA-II (197.3 ng/ml) and AFP (20.0 ng/ml) levels were significantly higher in the HCC subjects than in CLD or HC subjects (*p* < 0.05), a finding consistent with earlier reports [18, 32] but differed with Huang et al. [24] despite similar techniques and analysers. Again, the exact reasons are unclear and could be multifactorial.

Additionally, among the 33 subjects with confirmed HCC, 16 subjects (48.5%) demonstrated negative AFP levels. In contrast, 7 subjects (21.1%) were found to have negative PIVKA-II levels. Notably, 9 subjects (27.3%) exhibited a distinct biomarker profile characterized by AFP negativity with concomitant PIVKA-II positivity, indicating that a proportion of AFP-negative HCC cases could still be detected through PIVKA-II measurement in line with previous reports [16]. A statistically significant stepwise increases in GAAD score, PIVKA-II and AFP levels was observed among HCC, CLD, and HC subjects, with the highest values detected in HCC group, and lowest in HC. This stepwise gradient reflects progressive hepatocarcinogenesis, whereby malignant transformation is associated with increasing dysregulation of hepatic protein synthesis and vitamin K–dependent coagulation pathways [14, 33].

Interestingly, nearly half of the HCC subjects in the present cohort were AFP-negative despite many presenting with relatively large or multifocal tumours. This finding highlights the recognised biological heterogeneity of HCC and suggests that AFP alone may be insufficient for identifying a clinically important subset of Malaysian patients with HCC. The relatively high frequency of AFP-negative HCC observed in this study supports the need for complementary biomarkers such as PIVKA-II and multivariable diagnostic models such as GAAD, which integrate demographic and biochemical variables to improve diagnostic discrimination. Nevertheless, these findings require confirmation in larger prospective cohorts encompassing the full spectrum of HCC stages.

With regards to diagnostic performance, in differentiating HCC from CLD, the GAAD score demonstrated the highest diagnostic accuracy, with an AUC of 0.87 at the standard cutoff of 2.57, together with high sensitivity (81.82%) and NPV (80.0%), similarly reported by Asian studies [22, 31, 34, 35]. The relatively lower specificity of the GAAD score observed in this study compared to some Western cohorts may be attributed to elevated PIVKA-II levels among CLD subjects without HCC, particularly those with hepatitis B–related cirrhosis [14, 36]. The optimal cutoff level for PIVKA-II for differentiating HCC from CLD was 28.4 ng/ml (sensitivity 75.76%, specificity 69.70%), in agreement with other report [34].

Interestingly, AFP demonstrated an AUC of 1.000 despite a relatively low sensitivity of 51.5% at the conventional cutoff value of 20 ng/mL. This finding is not contradictory, as the AUC reflects the overall discriminatory capacity of a biomarker across the entire range of threshold values, whereas sensitivity and specificity are dependent on a single selected cut-off. The conventional AFP threshold prioritizes specificity at the expense of sensitivity, potentially missing a substantial proportion of HCC cases with lower AFP concentrations. Similar observations have been reported in previous studies, highlighting the limitations of relying solely on AFP at fixed clinical thresholds and supporting the use of complementary biomarkers such as GAAD and PIVKA-II to improve diagnostic sensitivity. Others reported 24–75% sensitivity [15, 34]. Notably, PIVKA-II outperformed AFP in sensitivity, also consistent with others [20, 37–40].

Although GAAD demonstrated excellent diagnostic performance in the present study, the near-perfect discrimination observed in some ROC analyses should be interpreted cautiously. The present investigation was conducted as a single-centre diagnostic accuracy study with predefined comparison groups, and therefore the observed performance may partly reflect spectrum effects (case–control separation) and the sample characteristics of the study population. Independent external validation in larger multicentre cohorts is required to determine the reproducibility and generalisability of these findings.

In differentiating HCC from HC subjects, the GAAD score demonstrated the highest sensitivity and NPV compared to PIVKA-II and AFP alone, consistent with findings reported by Huang et al. [24]. These findings suggest that the GAAD score may serve as a useful screening tool for excluding HCC, potentially reducing unnecessary further investigations and treatment.

Although GAAD demonstrated superior diagnostic performance compared with AFP and PIVKA-II individually, its clinical implementation requires further evaluation. Because GAAD incorporates age, sex, AFP and PIVKA-II, its improved discrimination may partly reflects integration of established predictors rather than the addition of an independent biomarker. The present study was designed to evaluate diagnostic accuracy rather than clinical testing strategies; therefore, subgroup analyses among AFP-negative or PIVKA-II-negative patients and formal cost-effectiveness analyses were beyond the scope of this work. Future prospective studies should investigate sequential testing approaches (e.g., AFP followed by GAAD in biomarker-negative patients versus direct GAAD testing provide greater clinical and economic benefit, and validate the model across different HCC risk populations and surveillance settings before widespread clinical implementation can be recommended.

This study has several limitations. First, it was conducted at a single centre with a relatively constrained sample size, which may limit the generalisability of the findings. While the sample size was calculated for the predefined three-group comparison, several secondary pairwise comparisons, which may have had relatively lower statistical power. Therefore, these findings should be interpreted cautiously and validated in larger multicentre studies. Second, biomarker performance was not assessed individually across each HCC stages or disease aetiologies or tumour burden, which may influence diagnostic accuracy. Third, the predominance of advanced-stage HCC in this cohort may have overestimated biomarker sensitivity. The HCC group contained a substantial proportion of patients with larger tumour burden, including tumours larger than 5 cm and multiple nodules. As advanced or bulky HCC may produce higher concentrations of tumour biomarkers, the diagnostic performance observed in this study may overestimate the sensitivity of GAAD, PIVKA-II and AFP in real-world surveillance settings, where the intended target is early-stage HCC. Therefore, the present findings should not be interpreted as definitive evidence for early HCC screening. Future prospective studies involving a larger proportion of early-stage HCC, including small solitary tumours, are required to validate the role of GAAD and related biomarkers in surveillance and early detection. Fourth, the present study evaluated serum biomarkers independently and did not assess their performance when combined with ultrasonography, which remains the recommended for HCC surveillance. Therefore, the findings should not be interpreted as evidence that GAAD or PIVKA can replace imaging-based surveillance. Prospective studies evaluating integrated biomarker-imaging strategies are warranted. Fifth, although GAAD showed improved discrimination, its incremental clinical value beyond AFP and PIVKA-II testing, including sequential testing strategies and cost-effectiveness, was not evaluated. Future prospective studies should determine whether sequential testing approaches (e.g., AFP followed by GAAD in biomarker-negative patients) or direct GAAD testing provide greater diagnostic and economic benefit, particularly across different HCC risk populations and surveillance settings. Finally, external validation in larger, multicentre cohorts with diverse HCC risk profiles is warranted.

## Conclusion

In conclusion, GAAD demonstrated superior diagnostic discrimination compared with AFP and PIVKA-II in this Malaysian cohort and represents a promising serum-based diagnostic model for HCC. However, because the study population predominantly comprised patients with relatively advanced disease, further prospective validation in larger multicentre cohorts, particularly among individuals with early-stage HCC undergoing routine surveillance, is required before GAAD can be recommended for widespread clinical implementation.

### Future direction

Future studies should validate the diagnostic performance of GAAD, PIVKA-II, and AFP in larger multicenter cohorts with diverse patient populations. Longitudinal studies are also warranted to evaluate their effectiveness in HCC surveillance, particularly among high-risk individuals with chronic liver disease. Furthermore, the establishment of standardized assay-specific cutoff values and assessment of cost-effectiveness will be important to facilitate the integration of these biomarkers into routine clinical practice. The potential prognostic role of these biomarkers in predicting treatment response, disease progression, and recurrence should also be explored.

## Data Availability

Data supporting the findings can be requested to corresponding author karyatee@usm.my for a reasonable consideration.

## Abbreviations

AASLD: American Association for the Study of Liver Diseases
AFP: Alpha-feto protein
APASL: Asian Pacific Association for the Study of the Liver
AUC: Area under the curve
CI: Confidence interval
CLD: Chronic liver disease
CT: Computer tomography
DCP: Des-carboxy-prothrombin
EASL: European Association for the Study of the Liver
ECLIA: Electro-chemiluminescence immunoassay
HBV: Hepatitis B virus
HC: Healthy controls
HCV: Hepatitis C virus
HCC: Hepatocellular carcinoma
IQR: Interquartile range
MAFLD: Metabolic dysfunction-associated fatty liver disease
MASLD: Metabolic dysfunction-associated steatotic liver disease
NPV: Negative predictive values (NPV)
PIVKA-II: Prothrombin induced by vitamin K absence or antagonist-II
PPV: Positive predictive values
ROC: Receiver operating characteristic
SD: Standard deviation
US: Ultrasonography
